# Pneumoperitoneum Secondary to Colonic Perforation in a Domestic Rabbit

**DOI:** 10.3390/ani16020198

**Published:** 2026-01-09

**Authors:** Margherita Romano, Stefano Esposito, Pierantonio Battiato

**Affiliations:** 1 Clinica Veterinaria Città di Catania, Viale Vittorio Veneto, 313, 95126 Catania, Italy; margheritarmn@gmail.com; 2 Clinica Veterinaria Strada Ovest, Strada di San Pelaio, 127, 31100 Treviso, Italy; esvetexotics@gmail.com; 3 Antech Imaging Services, 17672-B Cowan, Irvine, CA 92614, USA

**Keywords:** exotic pet medicine, lagomorph, small mammals, abdominal gas, intestinal rupture, case report

## Abstract

A two-year-old, intact female, domestic fawn-colored dwarf lop rabbit was presented with apathy and anorexia. Clinical examination revealed hypothermia, tachycardia, and abdominal pain. Routine hematology, biochemistry, radiographic and ultrasonographic examinations were performed. Radiographic evaluation demonstrated pneumoperitoneum, generalized small intestinal distention and marked focal dilation of the descending colon by amorphous and partially mineralized material. Ultrasonography confirmed the presence of free gas along with abdominal effusion. Exploratory laparotomy revealed focal perforation of the descending colon associated with mural abscess formation containing fecal material and pus. The severity of the lesions and multiple adhesions to adjacent organs (urinary bladder and uterus) led to the decision for intraoperative euthanasia. This case report describes clinical, imaging and surgical findings of pneumoperitoneum related to septic peritonitis secondary to colonic perforation in a domestic rabbit.

## Simple Summary

Pneumoperitoneum is defined as the presence of free gas within the abdominal cavity and, in domestic rabbits, represents a rare clinical condition. In most cases, it is secondary to gastrointestinal tract perforation, rapidly progressing to septic peritonitis with an unfavorable prognosis. This case report describes a pneumoperitoneum secondary to descending colon perforation in a domestic rabbit (*Oryctolagus cuniculus*). The aim is to contribute to the limited available literature and emphasize the importance of diagnostic imaging, particularly radiography, for early recognition and timely therapeutic management.

## 1. Introduction

Pneumoperitoneum in the domestic rabbit is rarely reported in the literature, with available data largely derived from experimental models [1,2,3,4,5,6,7].

In most reported cases in dogs and cats, the presence of free gas within the peritoneal cavity is associated with gastrointestinal perforation or penetrating trauma, although it may occasionally occur following diagnostic or surgical procedures [8,9,10,11,12,13,14]. In rabbit models, experimental pneumoperitoneum was associated with systemic effects on the liver, kidneys, and blood gas parameters [3,4,5,7]. In rabbits, gastrointestinal diseases are among the most common clinical presentations and include gastrointestinal stasis, mechanical obstruction (e.g., secondary to trichobezoar, phytobezoar), colonic obstruction secondary megacolon (in *English Spotting* Coat Color Locus rabbits) and acute gastric dilation [15,16,17,18,19,20,21,22], often related to motility dysfunction or chronic inflammatory processes affecting the distal tract, such as sacculitis and appendicitis [23]. Based on our review of the literature, no cases of spontaneous clinical pneumoperitoneum secondary to gastrointestinal perforation have been reported to date. Available reports describing free peritoneal gas are limited to experimental studies [1,2,3,4,5,6,7]. However, in small animals, gastrointestinal perforation with subsequent pneumoperitoneum represents an exceptional and potentially fatal event characterized by rapid progression to septic peritonitis and endotoxic shock [8,9,10,11]. Diagnosis of pneumoperitoneum requires a multimodal approach mainly based on diagnostic imaging: radiography remains the gold standard for identifying free peritoneal gas, while ultrasonography allows confirmation of ring down or reverberation artifacts and the assessment of associated effusions [11,24,25,26]. In rabbits, colonic perforation may be associated with intraluminal obstruction (e.g., foreign material such as trichobezoars or phytobezoars), often in the context of gastrointestinal stasis and dietary imbalances. Other possible etiologies include chronic inflammatory processes, neoplasia, or, less commonly, other foreign body ingestion [22,27]. This case report describes a rare instance of pneumoperitoneum secondary to perforation of the descending colon in a domestic rabbit, aiming to contribute to the limited available literature and to emphasize the importance of early diagnosis and prompt therapeutic intervention.

## 2. Case Description

A two-year-old, intact female, fawn-colored dwarf lop rabbit, weighing 2 kg, was presented with a 24 h history of apathy and anorexia. The rabbit was fed a commercial diet containing cereals, hay, and fresh vegetables. On physical examination, the patient presented with hypothermia (37.0 °C), tachycardia (300 bpm), and marked abdominal pain. The mucous membranes appeared mildly pale. Hematological analysis revealed heterophilic leukocytosis (WBC: 18.5 × 10^9^/L; heterophils 82%), mild normocytic normochromic anemia (PCV: 28%), and an increase in total protein (7.2 g/dL), findings consistent with systemic inflammation.

**Figure 1:**
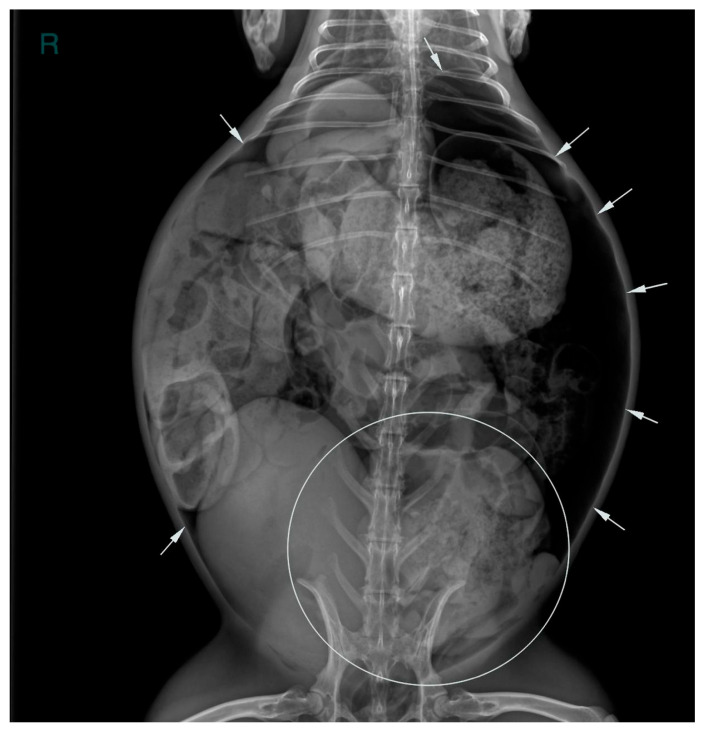
Ventrodorsal abdominal radiographic view (kVp: 50, mAs; 2.4); the ‘R’ marker indicates the right side of the abdomen. Marked distension of the descending colon due to amorphous, partially mineralized material (circle) and the presence of free gas within the peritoneal cavity (white arrows).

**Figure 2:**
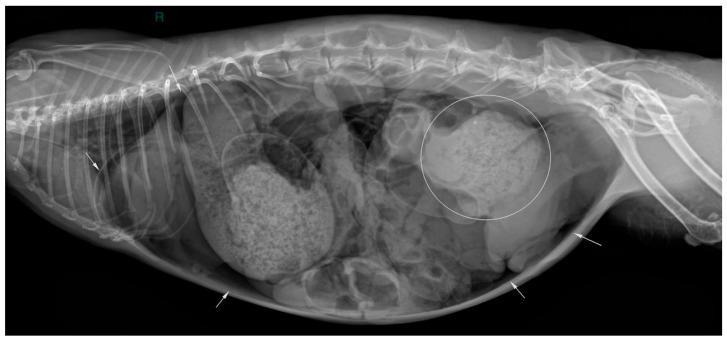
Right lateral abdominal radiographic view (kVp: 50, mAs; 2.4); the ‘R’ marker indicates right lateral positioning. Marked dilation of the descending colon by amorphous, partially mineralized material (circle) and evidence of free peritoneal gas (white arrows).

Two abdominal radiographic views of the abdomen were performed (right lateral and ventrodorsal views; kv 50 mAs 2.4; EXAMION GmbH X-DR L Wifi Gen 3, Fellbach, Germany). Large amounts of free peritoneal gas usually float to the highest point within the abdomen. As in this case, the presence of free peritoneal gas outlines serosal surfaces of the viscera, such as bowel loops, stomach, liver and diaphragm [26]. Abdominal radiographic examination revealed pneumoperitoneum with moderate generalized distension of the small intestinal loops and the presence of multifocal air–fluid levels. The cecum appeared poorly distended with gas, showing increased visibility of the haustra. The caudal aspect of the descending colon was focally markedly distended by amorphous, partially mineralized material, suggestive of an obstruction at that level (Figure 1 and Figure 2).

Ultrasonography (MyLabSix, Esaote, Genova, Italy) confirmed the presence of free gas within the peritoneal cavity, visualized as ring down and reverberation artifacts, associated with a mild hyperechoic effusion in the cranial and mid-abdominal quadrants.

Based on the diagnostic findings, a diagnosis of pneumoperitoneum secondary to presumptive suspected gastrointestinal perforation was made. Considering the marked distension of the descending colon with amorphous intraluminal material, this region was suspected to be the primary site of perforation.

The patient was initially stabilized using crystalloid fluid therapy (Lactated Ringer’s Solution, B. Braun Vet Care, Tuttlingen, Germany) with a resuscitation bolus of 5 mL/kg over 10 min, followed by a maintenance infusion of 8 mL/kg/h. To reduce intra-abdominal pressure, a decompressive paracentesis was performed using a 20-G needle. Pharmacological therapy included enrofloxacin (5 mg/kg IV q12h; Valemas, Ati, Bologna, Italy), metronidazole (5 mg/kg IV q12h; Metronidazolo Kabi, Fresenius Kabi, Verona, Italy), and meloxicam (1 mg/kg SC q24h; Metacam, Boehringer Ingelheim, Rhein, Germany).

Given the severity of the clinical condition, an exploratory laparotomy was performed. The anesthetic protocol consisted of premedication with dexmedetomidine (20 µg/kg IM; Sedadex, Dechra, Northwich, United Kingdom), ketamine (10 mg/kg IM; Nimatek, Dechra, Northwich, United Kingdom), and methadone (0.5 mg/kg IM; Semfortan, Dechra, Northwich, United Kingdom), followed by induction with propofol (1 mg/kg IV to effect; Propomitor, Orion Pharma, Espoo, Finland) and intubation using a 3.0 mm laryngeal tube. Anesthesia was maintained with a fresh gas flow of 0.5 L/min, using a non-rebreathing (Mapleson D) breathing system, with a mixture of oxygen and medical air (1:2 ratio) and isoflurane at a vaporizer setting of 2% (Vetflurane, Virbac, Carros, France). Monitoring included pulse oximetry, electrocardiography, and capnography, all initiated prior to the start of surgery.

**Figure 3:**
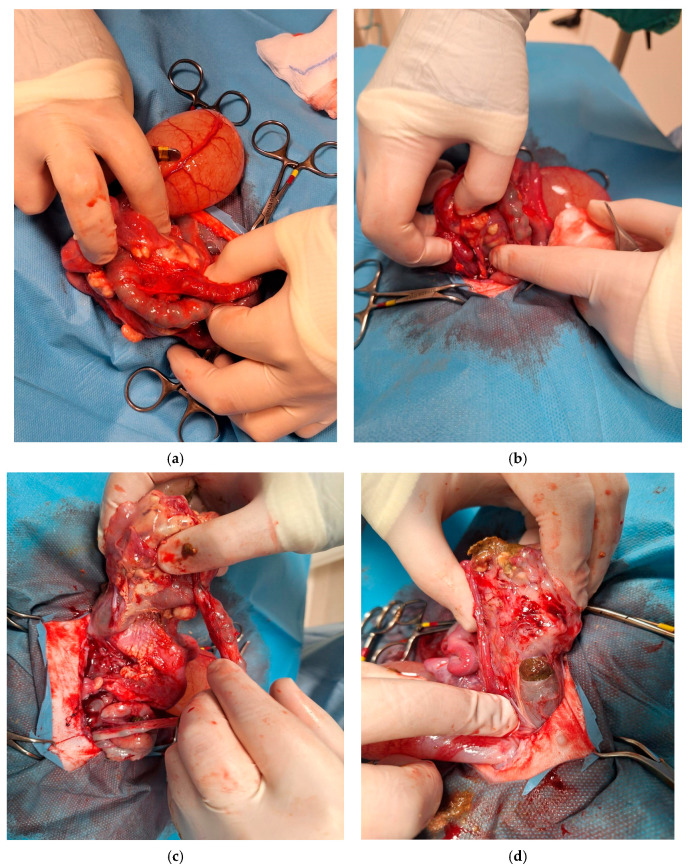
Sequence of intraoperative findings during surgery. (**a**) At laparotomy, an intestinal segment was found adherent to the uterine and cervical regions, forming adhesions between the distal rectal tract and adjacent abdominal structures. (**b**) During dissection of the intestinal tract and surrounding structures, abscess formation became evident and was carefully isolated from adjacent tissues to delineate its extent and dimensions. (**c**) Intraoperative visualization of the colic intestinal segment and the terminal portion of the descending colon, showing fecal accumulation and the development of an abscess beneath this area. (**d**) A fistulous tract can be observed originating from a lesion in the terminal portion of the colon. The tract extends through the mucosal, submucosal, and muscular layers, with evident fecal leakage and loss of fecal material.

Exploratory laparotomy revealed marked dilation of the descending colon, associated with mural abscess formation and a dorsal perforation of approximately 0.5 cm in diameter. The concurrent evidence of leakage of fecal material and pus was indicative of an ongoing parietal necrosis. A partial resection of the descending colon was undertaken; however, intra-operative exploration revealed extensive, dense adhesions involving multiple abdominal organs, with marked disruption of normal anatomical planes. Multiple fistulous communications were identified between the descending colon, urinary bladder, and uterus, consistent with a chronic, severe inflammatory process. Additionally, diffuse abdominal contamination and established septic peritonitis were present, indicating ongoing leakage of intestinal contents. The extent and chronicity of these lesions substantially limited the feasibility of further surgical intervention. Definitive surgical management would have required multiple, complex resections associated with a high risk of anastomotic dehiscence, persistent intra-abdominal sepsis, and severe postoperative complications. Given the widespread tissue involvement, compromised tissue integrity, and the presence of septic peritonitis, the likelihood of achieving functional gastrointestinal recovery and an acceptable postoperative quality of life was considered extremely low. Accordingly, in light of the grave prognosis for meaningful recovery despite aggressive surgical and intensive postoperative management, intraoperative euthanasia was elected. The main surgical stages are illustrated in Figure 3.

Histopathology has not been performed as the patient was euthanized during exploratory laparotomy, and no further final diagnostic procedures were requested or accepted by the owners. Possible etiologies, given the macroscopic findings (presence of purulent material with abscess formation), were mainly chronic inflammation with the presence of foreign material as a primary cause. No obvious masses or macroscopic evidence of neoplastic infiltration was noted during surgical exploration.

## 3. Discussion

Diagnosing pneumoperitoneum in rabbits represents a notable clinical challenge, as the physiological presence of gas within the gastrointestinal tract may complicate radiographic interpretation and make it difficult to differentiate between physiological and pathological conditions. In this species, published information regarding the diagnosis and management of pneumoperitoneum remains extremely limited. Consequently, the diagnostic approach in the present case was largely informed by data from canine and feline veterinary medicine, where the pathophysiology and imaging characteristics of pneumoperitoneum have been extensively described [11]. Non-traumatic pneumoperitoneum is mainly reported in dogs and cats, with etiologies commonly associated with gastric perforation, neoplasia, or idiopathic causes [8,9,10,12]. In these species, radiography is considered the diagnostic modality of choice for detecting free intraperitoneal gas. However, small gas volumes may be difficult to visualize on conventional vertical-beam radiographs because gas bubbles can overlap with visceral structures and mimic intraluminal gas. Free gas tends to accumulate in non-dependent regions of the abdomen; therefore, the use of horizontal-beam view and appropriate patient positioning (dorsal recumbency with slight cranial elevation) substantially increases diagnostic sensitivity [11,24,25,26]. Radiological and clinical studies in human and veterinary medicine have further validated the diagnostic accuracy of both radiography and ultrasonography in detecting small amounts of intraperitoneal gas, emphasizing the utility of horizontal-beam projections and the “enhanced peritoneal stripe sign” as a reliable ultrasonographic indicator of pneumoperitoneum [25,26,28,29]. In the present case, the radiographic identification of gas bubbles outside the intestinal lumen strongly suggested a perforation of the descending colon, as the presence of free intraperitoneal air in the absence of prior surgery or penetrating trauma is widely recognized as highly indicative of visceral rupture [26]. Ultrasonography proved to be a valuable complementary diagnostic tool, confirming the presence of free gas through ring down and reverberation artifacts and increased echogenicity of the peritoneal line (“enhanced peritoneal stripe sign”), while also revealing a small amount of echogenic peritoneal effusion and guiding decompressive paracentesis. Previous studies have demonstrated that ultrasonography can detect even minimal volumes of air (as little as 0.4 mL), although diagnostic sensitivity largely depends on operator experience [25]. Integration of radiographic and ultrasonographic findings, together with clinical evidence of progressive abdominal distension, enabled a definitive diagnosis of pneumoperitoneum secondary to intestinal perforation. As described in the canine and feline literature, this condition carries a substantial risk of septic peritonitis and requires prompt surgical intervention combined with intensive postoperative care to improve clinical outcomes [8,9,11,27]. Experimental studies in rabbits have further shown that carbon dioxide insufflation can induce significant cardiovascular, respiratory, and microcirculatory alterations [1,2,6,7], suggesting that sustained intra-abdominal pressure may contribute to intestinal ischemia and subsequent increased severity of wall necrosis and perforation [30]. In domestic rabbits, intestinal perforation and secondary pneumoperitoneum may also develop in the setting of underlying gastrointestinal dysfunction or obstruction. Common obstructive conditions in this species include trichobezoars and phytobezoars, which are frequently associated with inadequate dietary fiber intake, dehydration, and impaired gastrointestinal motility. Diet-related alterations, particularly diets low in indigestible fiber and relatively high in carbohydrates, are well-recognized predisposing factors for gastrointestinal hypomotility, dysbiosis, and luminal impaction. These conditions may result in progressive intestinal distension, increased intraluminal pressure, and compromised intestinal wall perfusion, thereby increasing the risk of mural ischemia, necrosis, and eventual perforation. Although no definitive obstructive material was identified in the present case, functional or partial obstruction related to gastrointestinal dysmotility cannot be excluded and may have contributed to intestinal compromise and the subsequent development of pneumoperitoneum [22].

Given the current scarcity of species-specific literature, the present case contributes to expanding the existing knowledge on pneumoperitoneum in domestic rabbits. It underscores the importance of a multimodal diagnostic approach, as well as the critical need for early recognition and timely intervention to improve prognosis in this rare but potentially life-threatening condition. Histopathological examination or necropsy remains pivotal for definitively identifying the underlying cause of gastrointestinal mural perforation resulting in pneumoperitoneum.

## 4. Conclusions

The present report describes a rare case of pneumoperitoneum secondary to perforation of the descending colon in a domestic rabbit. Radiography remains the imaging modality of choice for identifying free peritoneal gas, also in domestic rabbits, while ultrasonography serves as a valuable complementary tool for diagnostic confirmation and assessment of associated peritoneal effusion. The clinical experience reported here reinforces the importance of early imaging evaluation, careful radiographic and ultrasonographic interpretation, and timely surgical intervention in cases of suspected colonic perforation with related pneumoperitoneum. This report documents a rare presentation of pneumoperitoneum secondary to gastrointestinal perforation in a domestic rabbit and contributes to the limited clinical literature on this condition, with particular emphasis on its diagnostic features.

## Data Availability

Data are contained within the article.

## Abbreviations

The following abbreviations are used in this manuscript:

Bpm	Beats per minute
CO_2_	Carbon Dioxide
PCV	Packed Cell Volume
WBC	White Blood Cells
IV	Intravenous
IM	Intramuscular
SC	Subcutaneous
Q12h	Every 12 h
Q24h	Every 24 h
